# Prevalence and pattern of waterborne parasitic infections in eastern Africa: A systematic scoping review

**DOI:** 10.1016/j.fawpar.2020.e00089

**Published:** 2020-09-08

**Authors:** Helena A. Ngowi

**Affiliations:** Department of Veterinary Medicine and Public Health, Sokoine University of Agriculture, P.O. Box 3021, Morogoro, Tanzania

**Keywords:** Waterborne, Parasitic-diseases, Eastern Africa, Burden

## Abstract

Waterborne parasitic diseases form one of common and important public health and economic problems in low- and middle-income countries, though little is known on the burden and patterns of these diseases in most regions. This systematic scoping review informs on the prevalence and pattern of waterborne parasitic infections in eastern Africa from 1st of January 1941 to 31st of December 2019. The review found limited number of published studies on waterborne parasitic diseases, though 13 of the 15 studied countries in eastern Africa provided one or more published report(s) totalling 47 reports. Focus of studies was mainly on schistosomiasis where 44.8% of the 47 retrieved studies reported it. Other frequently reported diseases were giardiasis (23.4% of reports), soil-transmitted helminths (23.4%) and amoebiasis (21.3%). Rarely reported diseases were malaria, cryptosporidiosis, isosporiasis, dracunculiasis and trichomoniasis. Based on parasitological examinations, schistosomiasis prevalence ranged from 17 to 33% in Burundi, 1.9 to 73.9% in Ethiopia, 2.1 to 18% in Kenya, 7.2 to 88.6% in Uganda, 22.9 to 86.3% in Tanzania, 27.2 to 65.8% in Somalia, 15 to >50% in Mauritius, 2.4% in Eritrea and 5.0 to 93.7% in Madagascar. Amoebiasis prevalence was 4.6–15,3% (Ethiopia), 5.9–58.3% (Kenya), 54.5% (Rwanda), 0.7–2.7% (Sudan), 19.93% (Uganda) and 4.5–5.0% (Seychelles). Giardiasis prevalence was 0.6–55.0% (Ethiopia), 16.6% (Kenya), 3.6% (Rwanda), 21.1% (Sudan), 40.7% (Uganda), 45.0% (Eritrea) and 3.3–6.0% (Seychelles). Soil-transmitted helminths prevalence was 41.7–52.4% (Ethiopia), 32.4–40.7% (Kenya), 9997 cases (Rwanda), 85.0% (Somalia), 4.7% (Madagascar) and 1.1–84% (Seychelles), *Ascaris lumbricoides*, *Trichuris trichiura* and hookworms were the most common helminths detected. Malaria prevalence was 2.9–4.31% (Ethiopia), an annual episode of 9 million people (Sudan), 13.0% (Tanzania), 146 hospital cases (Madagascar), 1.4–2.0% (Seychelles) and <5.0% in Djibouti. It is also observed that >50% of the populations in eastern Africa region lack improved drinking water sources or sanitation facilities. This may account for the observed high prevalence of the diseases. The author also suggests likely underestimation of the prevalence as most waterborne parasitic diseases are neglected and cases likely only recorded and left unpublished in health facilities. Thus for a thorough mapping of burdens of these diseases, grey literature, including hospital records must be reviewed while interventions focusing on improved water and sanitation are likely to reduce the burden considerably.

## 1 Introduction

Waterborne diseases are diseases and infections caused by pathogenic microorganisms or toxic substances found in water, which may lead to ill health. A broader definition includes diseases linked to water scarcity or contamination and diseases related to vectors whose part of their life cycle occurs in water habitats (Stanwell-Smith, 2010). Infections can be acquired while bathing, washing, drinking water, eating food exposed to contaminated water, or by being bitten by an infected vector such as in the case of malaria. Classic waterborne diseases are those predominantly transmitted through contact with or consumption of contaminated water. Waterborne parasitic diseases are commonly caused by pathogenic protozoa and helminths. Worldwide, a number of parasite species are known to cause waterborne infections, including protozoa: *Entamoeba* spp. (causing amoebiasis), *Cryptosporidium* spp. (cryptosporidiosis), *Giardia* spp. (giardiasis) and helminths (particularly *Schistosoma* spp., *Dracunculus medinensis* and a number of soil-transmitted nematodes such as *Ascaris* and *Trichuris* species).

In addition to public health impact, waterborne diseases can have a significant impact on the economy of endemic countries and globally. While there are global concerns regarding waterborne diseases, especially in resource poor countries such as those of Africa, information on the burden and distribution of these diseases is limited. This information is required to guide control efforts to safeguard public health and improve well being. The present systematic scoping review summarizes prevalence and patterns of waterborne parasitic diseases in eastern Africa to guide national and global efforts to control the diseases.

## 2 Materials and methods

### 2.1 Study area

**Fig. 1:**
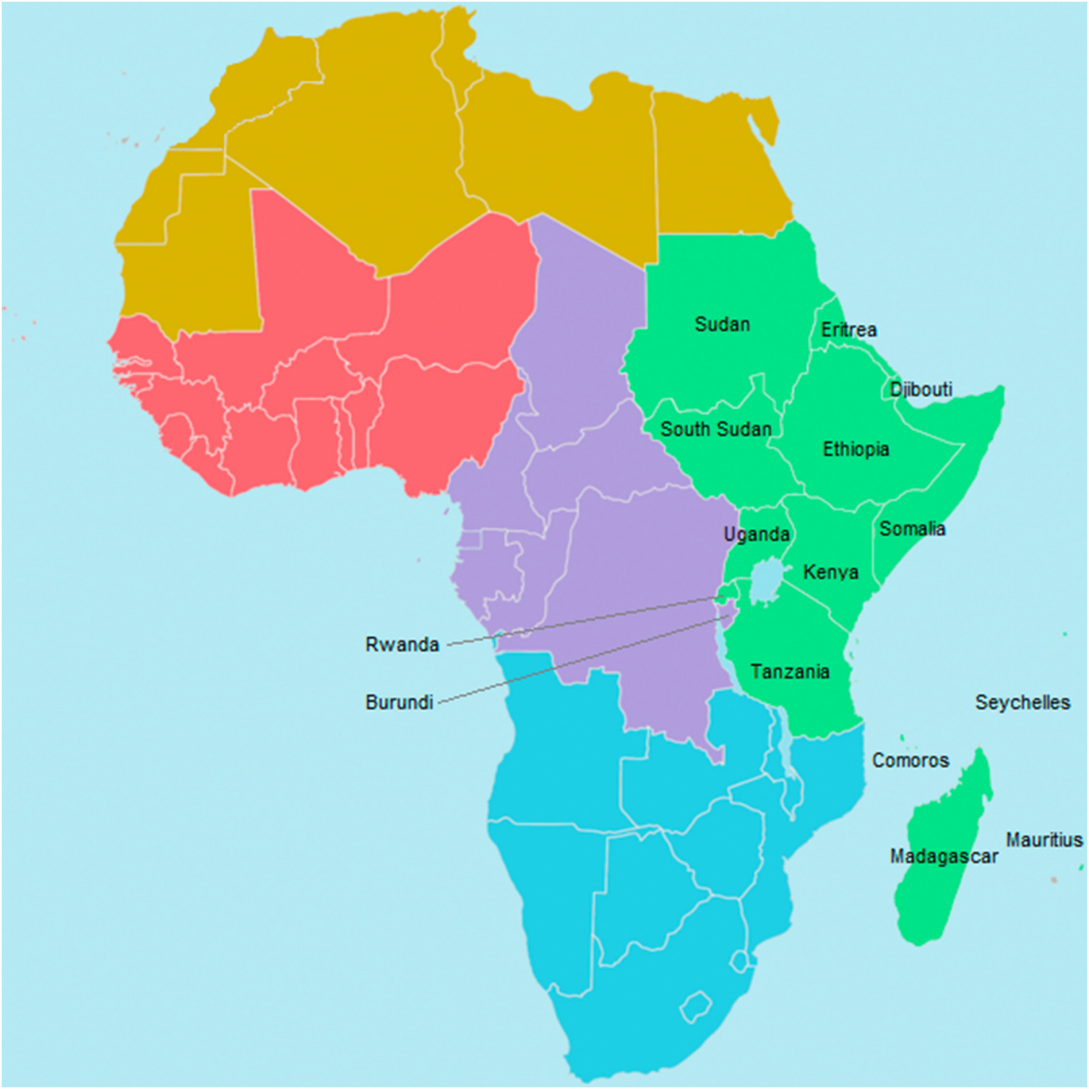
Regions of the AU: . The study area was eastern Africa, including Burundi.

Source: Ali A. Fazal, CC BY-SA 3.0, https://commons.wikimedia.org/w/index.php?curid=30922828.

Eastern Africa has been defined in different ways by different bodies and organisations for various reasons. The present study adopted a definition by African Union (Fig. 1) with slight modification by including Burundi in the review. Thus 15 countries were included, namely, Sudan, South Sudan, Eritrea, Djibouti, Ethiopia, Somalia, Uganda, Kenya, Burundi, Rwanda, Tanzania, Madagascar, Comoros, Mauritius and Seychelles. The literature search was performed between March 25 and May 24, 2020 and included studies published from January 1, 1941 to December 31, 2019 as elaborated below.

### 2.2 Study design

This was a systematic scoping review.

### 2.3 Inclusion criteria for studies

#### 2.3.1 Types of participants (studies)

This review included published reports of waterborne parasitic diseases in humans in the eastern Africa countries.

#### 2.3.2 Concept

The review included any published work conducted to measure prevalence, incidence or morbidity of parasitic diseases that can be acquired by human directly or indirectly. These include illnesses related to water shortage or water contamination for any reason and diseases related to vectors with part of their life cycle in water habitats (Nwabor et al., 2016).

#### 2.3.3 Context

This review was intended to map magnitude of waterborne parasitic diseases in eastern Africa. Therefore, the review focused on the 14 countries of eastern Africa as defined by African Union (Fig. 1) and added Burundi because of its spatially well located in eastern Africa and the fact that the United Nations also define Burundi as an East African country. Thus 15 countries were included. The review included studies published from 1st of January 1941 to 31st of December 2019 based on an assumption that countries would conduct their first scientific research after they got independence from colonialism. For eastern Africa region, Ethiopia was the first to become independent, which was on 5th of May 1941.

**Fig. 2:**
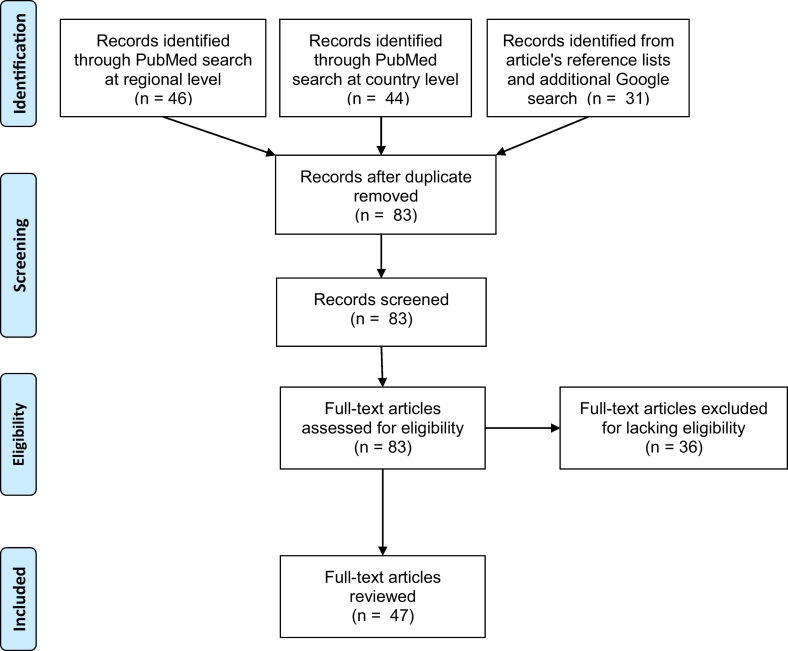
PRISMA diagram for a systematic scoping review on prevalence and pattern of waterborne parasitic infections in eastern Africa. 2019.

### 2.4 Searching strategy

Literature searches followed the standard three-steps described in the Joanna Briggs Institute (JBI) guidelines (Peters et al., 2015) with an additional fourth step. In the first step, a decision was made on which databases to search. Based on initial searches, PubMed was found to be the most common database that captured most of the articles in this research topic. The second step was searching the database for articles. A full description of the PubMed search is presented in Appendix A. The third step was searching additional relevant articles as could be found from reference lists of the primary articles retrieved in the second step. The fourth step was a general Google search of published records by country and disease, especially for countries that did not reveal any record from the previous three search steps. Searching for published reports on malaria was done for all 15 countries having revealed no record from the previous search steps. To maximise article capture, the PubMed search was performed using two approaches (see Appendix A for details). The first approach was searching the disease information at regional level (eastern Africa). The second approach was searching information at country level to allow possible retrieval of additional literature just in case a member country was not well cited as being an eastern African country. Fig. 2 presents the Preferred Reporting Items for Systematic Reviews and Meta-Analyses (PRISMA) flow diagram for this study.

### 2.5 Extracting and charting the results

Data extraction and charting was performed as described by Peters et al. (2015). Basic information extracted from each article is summarized in Appendix B. This include study country, study year, study population and sample size, parasite/disease studied, diagnostic test used, prevalence of infection and article reference.

## 3 Results

### 3.1 General results

A total of 15 countries of eastern Africa were reviewed in relation to waterborne parasitic diseases. Published reports on waterborne parasitic diseases were found in 13 (86.7%) of the countries. These together provided a total of 47 published reports (Table 1). Ethiopia carried 27.7% of the studies, followed by Kenya (12.8%) and Uganda (10.6%). The earliest publication was from 1953, found in Mauritius, while the latest publications were for 2019 in Ethiopia (three studies). No publication was retrieved from South Sudan or Comoros relevant for this study. The only two reports retrieved from Sudan were published in 1995 and 2007 when South Sudan was still part of Sudan as South Sudan gained its independence from Sudan on 9th of July 2011. Thus South Sudan cannot be disentangled from the disease prevalence reported in Sudan in this study.

**Table 1:** Number of records on waterborne parasitic diseases by country in eastern Africa between January 1941 and of December 31, 2019.

Country	Human population (UN 1 2019)	Total records found	Schistosomiasis	Amoebiasis	Giardiasis	Cryptosporidiosis	Isosporiasis	Dracunculiasis	Trichomoniasis	STHs	Malaria
Sudan	42,813,238	2		1	1						1
Ethiopia	112,078,730	13	5	4	4	3	1	1		2	1
Uganda	44,269,594	5	3	1	1						
Kenya	52,573,973	6	3	1	1					3	
Rwanda	12,626,950	2		1	1				1	2	
Burundi	10,864,245	1	1								
Tanzania	58,005,463	4	2			1					1
South Sudan	11,062,113										
Eritrea	3,497,117	3	2		1						
Djibouti	973,560	1									1
Somalia	15,442,905	3	2							1	
Madagascar	26,969,307	3	2							1	1
Comoros	850,886										
Mauritius	1,198,575	1	1								
Seychelles	97,739	3		2	2					2	1
**Total**	**393,324,395**	**47**	**21**	**10**	**11**	**4**	**1**	**1**	**1**	**11**	**6**

STHs - soil-transmitted helminths.

### 3.2 Prevalence of waterborne parasitic diseases in eastern Africa

Nine (9) of the 15 countries studied reported prevalence of schistosomiasis. This was followed by giardiasis (7), amoebiasis, STHs and malaria (6 each). Only one or two countries reported cryptosporidiosis, isosporiasis, dracunculiasis or *Trichomonas intestinalis* (Table 1). More details on each study are provided in Appendix B. Various diagnostic tests as well as categories of study populations were used by the studies. Therefore, comparison of the levels of infections between countries or studies must be made with caution.

#### 3.2.1 Schistosomiasis

Prevalence of schistosomiasis has been reported in eastern Africa based on parasitological examination using Kato-Katz method mostly. Prevalence has been estimated ranging from 17 to 33% in Burundi (Gryseels, 1991), 1.9 to 73.9% in Ethiopia (Wondimagegnehu et al., 1992; Abebe et al., 2014; Nyantekyi et al., 2014; Tadege and Shimelis, 2017; Shumbej and Girum, 2019), 2.1 to 18% in Kenya (Handzel et al., 2003; Mwandawiro et al., 2013; Won et al., 2017), 7.2 to 88.6% in Uganda (Kabatereine et al., 1992; Kabatereine et al., 2004; Tukahebwa et al., 2013), 22.9 to 86.3% in Tanzania (Poggensee et al., 2005; Kaatano et al., 2015). Schistosomiasis has also been reported in Somalia (27.2–65.8%) (Arfaa, 1975; Koura et al., 1981), Mauritius (15 > 50%) (Cowper, 1953), Eritrea (2.4%) (Ministry of Education, 2002; Lai et al., 2015) and Madagascar (5.0–93.7%) (Rasoamanamihaja et al., 2016; Spencer et al., 2017).

#### 3.2.2 Amoebiasis

Amoebiasis was also diagnosed mostly using Kato-Katz method. Prevalence estimates ranged from 4.6 to 15,3% in Ethiopia (Gedle et al., 2017; Gadisa and Jote, 2019; Sisay and Lemma, 2019), 5.9 to 58.3% in Kenya (Kipyegen et al., 2012), 54.5% in Rwanda (Niyizurugero et al., 2013), 0.7 to 2.7% in Sudan (Karrar and Rahim, 1995), 19.93% in Uganda (Ekou et al., 2012) and 4.5 to 5.0% in Seychelles (Kitua et al., 1988; Albonico et al., 1996).

#### 3.2.3 Giardiasis

Giardiasis mostly diagnosed using Kato-Katz and PCR techniques was reported in Ethiopia (0.6–55.0%) (de Lucio et al., 2016; Gedle et al., 2017; Gadisa and Jote, 2019; Sisay and Lemma, 2019), Kenya (16.6%) (Kipyegen et al., 2012), Rwanda (3.6%) (Niyizurugero et al., 2013), Sudan (21.1%) (Karrar and Rahim, 1995), Uganda (40.7%) (Johnston et al., 2010), Eritrea (45.0%) (Srikanth and Naik, 2004), and Seychelles (3.3–6%) (Kitua et al., 1988; Albonico et al., 1996).

#### 3.2.4 Soil-transmitted helminths

Soil-transmitted helminths have been diagnosed commonly using Kato-Katz technique. Prevalence have ranged from 41.7 to 52.4% in Ethiopia (Emana et al., 2015; Tadege and Shimelis, 2017), 32.4 to 40.7% in Kenya (Mwandawiro et al., 2013; Davis et al., 2014; Freeman et al., 2015), 9997 cases in Rwanda (Stone and Ndagijimana, 2018), 85.0% in Somalia (Ilardi et al., 1987), 4.7% in Madagascar (Rasoamanamihaja et al., 2016) and 1.1 to 84% in Seychelles (Kitua et al., 1988; Albonico et al., 1996). In all countries, *Ascaris lumbricoides*, *Trichuris trichiura* and hookworms were the most common helminths detected.

#### 3.2.5 Malaria

Most of malaria reports were based on retrospective hospital record surveys with some indication that diagnoses were based mostly based on blood smear examination. Prevalence of malaria was estimated at 2.9–4.31% in Ethiopia (Taffese et al., 2018), an annual episode of 9 million people in Sudan (Abdalla et al., 2007), 13.0% in Tanzania (Khatib et al., 2018), 146 hospital cases in Madagascar (Schlagenhauf et al., 2018), 1.4–2.0% in Seychelles (Ihantamalala et al., 2018) and <5.0% in Djibouti (Ollivier et al., 2011).

#### 3.2.6 Other parasitic diseases with few reports

A few diseases were reported by one or two of the 15 countries studied. The diseases include cryptosporidiosis reported in Ethiopia based on PCR (4.6–26.9%) (Adamu et al., 2014; de Lucio et al., 2016; Gedle et al., 2017) and Tanzania (4.3%) (Parsons et al., 2015), Others include Isosporiasis in Ethiopia (2.8%) (Gedle et al., 2017), dracunculiasis in Ethiopia (21 cases) (Jemaneh and Taticheff, 1993) and trichomoniasis in Rwanda (20.0%) (Niyizurugero et al., 2013).

## 4 Discussion

For the first time, published reports on waterborne parasitic diseases in eastern Africa have been collected and summarized. Articles written purely in other languages than English may not have been found, although no language restriction was put during the literature search.

Based on the information in this systematic scoping review, 86.7% of the countries in eastern Africa were the subject of investigations in that reported one or more types of waterborne parasitic disease(s) between 1953 and 2019. No published reports were found in the years from 1941 and 1952. The observed lack of investigations in South Sudan and Comoros does not mean absence of the diseases, but may reflect the parochial interest of investigators in the respective countries. In addition, political instabilities in some countries may also have hindered research progression in those countries. For example in this study, countries like Burundi, Somalia and Sudan could only provide old research reports (1970s to 1990s) possibly because of frequent political instabilities leading to lack of supportive environments for research. It has also been observed that, the number of studies reported in the countries represented in published work was unfortunately too small for the 78-year period analysed. This emphasizes the limitations of investigations following the interests of uncoordinated investigations in describing broad-scale disease patterns of regional importance by researchers on the diseases. Taking an example of Tanzania (the author's country), hospital records frequently show detection of some of parasitic diseases, notably, giardiasis, amoebiasis and STHs, none of which was revealed in the literature for this country. It was also surprising to find limited number of studies on the morbidity of malaria in eastern Africa, an endemic region. This could be partly explained by the fact that, in response to global effort on malaria control, many countries have focused on interventions rather than epidemiological investigations. Findings of the present study are consistent with those of a review study elsewhere, which found significant underreporting and underestimation of the true extent of water-related diseases in the pan-European region (WHO, 2016). This particular study warns that, available data from routine surveillance systems and reported through formal reporting channels represent only a small fraction of the total amount of disease occurring in the population (WHO, 2016).

The observed more research on schistosomiasis could be explained partly by its high endemicity. Of the diseases reported in this review, schistosomiasis and dracunculiasis are the only globally prioritised neglected tropical diseases (NTDs) (WHO, 2014). Thus the observed more studies on schistosomiasis can also be due to the fact that, it is acknowledged globally and in many countries as an important NTD through global support for research and control. On the other hand, dracunculiasis was reported by only Ethiopia despite being a global priority NTD (WHO, 2014). The finding from this review is consistent with the WHO's report on the status of dracunculiasis eradication and global distribution for 1980–2019, which shows that several countries had achieved elimination during the past decade, including Kenya, Uganda and Sudan while some countries including Ethiopia were still reporting cases until 2017 although at a decreasing trend (WHO, 2019).

Despite the small number of studies conducted on waterborne parasitic diseases in eastern Africa over the past seven decades, available information highlights that, prevalence of important parasitic diseases is still high in recent years. These include schistosomiasis (Rasoamanamihaja et al., 2016; Tadege and Shimelis, 2017; Won et al., 2017; Spencer et al., 2017 Shumbej and Girum, 2019), amoebiasis (Ekou et al., 2012; Niyizurugero et al., 2013; Gedle et al., 2017; Gadisa and Jote, 2019; Sisay and Lemma, 2019), giardiasis (Kipyegen et al., 2012; de Lucio et al., 2016; Gedle et al., 2017; Gadisa and Jote, 2019; Sisay and Lemma, 2019) and STHs (Mwandawiro et al., 2013; Davis et al., 2014; Freeman et al., 2015; Emana et al., 2015; Tadege and Shimelis, 2017; Stone and Ndagijimana, 2018). Most of these diseases are transmitted in poor water and sanitation environments. In the eastern Africa region, proportion of populations using improved drinking water sources are <50% to 75% while <50% of the populations have improved sanitation facilities (WHO, 2015). This may account for the observed high prevalence of waterborne parasitic diseases. Thus improving water and sanitation facilities would reduce the burden of these diseases. A systematic review and meta-analysis of studies reporting schistosome infection rates in people who do or do not have access to safe water and adequate sanitation revealed that, increasing access to safe water and adequate sanitation are important measures to reduce the odds of schistosome infection although most of the studies were observational and quality was poor (Grimes et al., 2014). It is presumed that most of the African regions will share similar experiences regarding the diseases except where differences in climatic conditions affect survival of particular parasites. Strengthening of water sanitation and hygiene (WASH) programmes in eastern Africa is particularly advocated to enhance control of waterborne parasitic diseases.

## 5 Conclusions

This literature review has found limited number of published studies on waterborne parasitic diseases in eastern Africa over the period of 78 years assessed. Nevertheless, available studies reveal high prevalence of schistosomiasis, amoebiasis, giardiasis and soil-transmitted helminths in the region even in recent years. For a thorough mapping of prevalence of these neglected tropical diseases, there is a need to include grey literature such as hospital records. Control of waterborne parasitic diseases should include improvement of drinking water sources and sanitation facilities. Introduction and strengthening of water sanitation and hygiene (WASH) programmes in communities and schools is particularly advocated.

The following are the supplementary data related to this article.

Appendix A Search strategy used during literature review on waterborne parasitic diseases in eastern Africa. Appendix A

Appendix B Characteristics of studies reviewed on prevalence and pattern of waterborne parasitic infections in eastern Africa. 2019. Appendix B

## Declaration of competing interest

The authors declare that they have no known competing financial interests or personal relationships that could have appeared to influence the work reported in this paper.
