# Supplementary material for: Prevalence and pattern of waterborne parasitic infections in eastern Africa: A systematic scoping review
Source: Food Waterborne Parasitol. 2020 Sep 8;20:e00089. doi: 10.1016/j.fawpar.2020.e00089 (PMC7508703; doi:10.1016/j.fawpar.2020.e00089)
Supplement: Appendix A — Search strategy used during literature review on waterborne parasitic diseases in eastern Africa. [file mmc1.pdf]

Appendix A: Search strategy used during literature review on waterborne parasitic diseases in eastern Africa.

<table>
<tr><td colspan="2">(a) Database: PubMed
(b) Date of search: 25th March 2020.
c) Limits: 1st January 1941 to 31st December 2019</td></tr>
<tr><td>(d) Regional level search terms:
Medical Subject Headings (MeSH) of the following terms and their entry terms (shown in bullets):

<u>Waterborne Diseases</u>
• Disease, Waterborne
• Diseases, Waterborne
• Waterborne Disease
• Water-related Diseases
• Disease, Water-related
• Diseases, Water-related
• Water related Diseases
• Water-related Disease

<u>Parasitology [Subheading]</u>
• protozoology
• protozoa
• helminthology</td><td>(e) Search 1 - Regional level

1) <u>Waterborne Diseases</u> [MeSH] (2205 items)
2) Disease, Waterborne (3228 items)
3) Diseases, Waterborne (2205 items)
4) Waterborne Disease (3228 items)
5) Water-related Diseases (9212 items)
6) Disease, Water-related (11507 items)
7) Diseases, Water-related (9212 items)
8) Water-related Disease (11507 items)
9) <u>Parasitology</u> [MeSH) (222421 items)
10) Protozoology (222939 items)
11) Protozoa (230542 items)
12) Helminthology (222682 items)
13) Parasites (258933 items)
14) <u>Africa, Eastern</u> [MeSH] (68227 items)
15) East Africa (79005 items)
16) Eastern Africa (68227 items)
17) British Indian Ocean Territory (68238 items)
18) <u>Prevalence</u> [MeSH] (2703772 items)
19) Prevalences (694815 items)</td></tr>
</table>

• parasites<br><br>Africa, Eastern<br>• East Africa<br>• Eastern Africa<br>• British Indian Ocean Territory<br><br>Prevalence<br>• Prevalences<br><br>Incidence<br>• Incidences<br><br>Morbidity<br>• Morbidities	20) Incidence [MeSH] (2868467 items)<br>21) Incidences (851690 items)<br>22) Morbidity [MeSH] (2714314 items)<br>23) Morbidities (883070 items)<br>24) 1 OR 2 OR 3 OR 4 OR 5 OR 6 OR 7 OR 8 (16615 items)<br>25) 9 OR 10 OR 11 OR 12 OR 13 (266652 items)<br>26) 14 OR 15 OR 16 OR 17 (79014 items)<br>27) 18 OR 19 OR 20 OR 21 OR 22 OR 23.(3370880 items)<br>28) 24 AND 25 AND 26 AND 27 (**54 items**)<br>With filters below (**46 items**)<br><br>Filters:<br>**Article types:**<br>• Clinical Study<br>• Clinical Trial<br>• Review<br>• Systematic Reviews<br><br>**Publication date:**<br>January 1, 1941. The first country (Ethiopia) got independence May 5, 1941.<br><br>*Overall regional level model (step 28) in details*<br>(((((((((((Waterborne Diseases) OR Disease, Waterborne) OR Diseases, Waterborne) OR Waterborne Disease) OR Water-related Diseases) OR Disease, Water-related) OR Diseases, Water-related)

	OR Water related Diseases) OR Water-related Disease)) AND (((((Parasitology) OR protozoology) OR protozoa) OR helminthology) OR parasites)) AND ((((Africa, Eastern) OR East Africa) OR Eastern Africa) OR British Indian Ocean Territory)) AND (((((Prevalence) OR Prevalences)) OR ((Incidence) OR Incidences)) OR ((Morbidity) OR Morbidities))
**(f) Country level searching**<br>Using same terms for disease as in (d) above but replace the region terms with country level terms:<br><br>Eritrea [MeSH]<br>Sudan [MeSH]<br>• Republic of the Sudan<br>South Sudan [MeSH]<br>Djibouti [MeSH]<br>• Somaliland, French<br>• Republic of Djibouti<br>• French Somaliland<br>Ethiopia [MeSH]<br>• Federal Democratic Republic of Ethiopia<br>Somalia [MeSH]<br>Uganda [MeSH]<br>• Republic of Uganda	**(g) Search 2 - Country level**<br>*Overall country level model in details and filtered as for regional model:*<br>((((((((((((((((((((((((((((((((Eritrea) OR Sudan) OR Republic of the Sudan) OR South Sudan) OR Djibouti) OR Somaliland, French) OR Republic of Djibouti) OR French Somaliland) OR Ethiopia) OR Federal Democratic Republic of Ethiopia) OR Somalia) OR Uganda) OR Republic of Uganda) OR Kenya) OR Republic of Kenya) OR Tanzania) OR United Republic of Tanzania) OR Zanzibar) OR Tanganyika) OR Rwanda) OR Republic of Rwanda) OR Ruanda) OR Burundi) OR Republic of Burundi) OR Urundi) OR Madagascar) OR Malagasy Republic)))) OR (((((((Comoros) OR Iles Comores) OR Comoro Islands) OR Mayotte) OR Mauritius) OR Agalega Islands) OR Seychelles))) AND ((((((Prevalence) OR Prevalences)) OR ((Incidence) OR Incidences)) OR ((Morbidity) OR Morbidities)))) AND ((((((((((((Waterborne Diseases) OR Disease, Waterborne) OR Diseases, Waterborne) OR Waterborne Disease) OR Water-related Diseases) OR Disease, Water-related) OR Diseases, Water-related) OR Water related Diseases) OR Water-related Disease)))) AND

Kenya [MeSH]<br>• Republic of Kenya<br>Tanzania [MeSH]<br>• United Republic of Tanzania<br>• Zanzibar<br>• Tanganyika<br>Rwanda [MeSH]<br>• Republic of Rwanda<br>• Ruanda<br>Burundi [MeSH]<br>• Urundi<br>• Republic of Burundi<br>Madagascar [MeSH]<br>• Malagasy Republic<br>Comoros [MeSH]<br>• Iles Comores<br>• Comoro Islands<br>• Mayotte<br>Mauritius [MeSH]<br>• Agalega Islands<br>Seychelles [MeSH]	((((((Parasitology) OR protozoology) OR protozoa) OR helminthology) OR parasites)))= **44 items**<br><br>**(h) After removal of overlaps [merging (e) with (g)] = 52 items**<br><br>**(i) After removal of ineligible articles [from (h)] = 16 items**<br><br>**(j) Articles searched from reference lists of the 16 articles = 10 items**<br><br>Thus a total of 26 articles were reviewed.
