# Supplementary material for: Prevalence and pattern of waterborne parasitic infections in eastern Africa: A systematic scoping review
Source: Food Waterborne Parasitol. 2020 Sep 8;20:e00089. doi: 10.1016/j.fawpar.2020.e00089 (PMC7508703; doi:10.1016/j.fawpar.2020.e00089)
Supplement: Appendix B — Characteristics of studies reviewed on prevalence and pattern of waterborne parasitic infections in eastern Africa. 2019. [file mmc2.pdf]

Appendix B. Characteristics of studies reviewed on prevalence and pattern of waterborne parasitic infections in eastern Africa. 2019.

Article S/No.	Study country	Study year	Study population and sample size	Parasite(s)/Disease(s) studied	Diagnostic test	Prevalence estimates (%), otherwise, stated	Reference
1	BURUNDI		General population	*Schistosoma mansoni*	Kato-Katz	17, 19, 26, 33 four areas	Gryseels 1991
2	ETHIOPIA	2015/2016	323	*Entanmoeba histolytica/*dispar trophozoite<br>*E. histolytica*/dispar cyst<br>*Giardia lamblia* trophozoite<br>*G. lamblia* cyst<br>*Cryptosporidium parvum*<br>*Isospora belli*		7.1<br>4.6<br>4.8<br>0.6<br>5.9<br>2.8	Gedle et al.2017

3		2009/2011	520 HIV/AIDS patients	*Cryptosporidium pavum*	PCR-RFLP	26.9	Adamu et al., 2014
4		2011/2012	371 family members	*Schistosoma mansoni*	Kato-Katz	73.9	Abebe et al., 2014
5			Village-to-village search for cases	Dracunculiasis		21 cases	Jemaneh and Taticheff 1993
6			311 banana plantation residents	*Schistosoma mansoni*<br>*Entamoeba histolytica*		1.9<br>0.6	Wondimagegnehu et al., 1992
7		2018	561 under-five children	*Entamoeba histolytica/dispar*<br>*Giardia lamblia*		15.3<br>10.2	Gadisa and Jote 2019
8		2016	Schoolchildren	*Entamoeba histolytica/dispar*<br>*Giardia lamblia*		5.21<br>4.69	Sisay and Lemma 2019
9		2017	597 school-aged children	*Schistosoma mansoni*	Kato-Katz	12.9	Shumbej and Girum 2019

10		2008	491 adult participants	Schistosomiasis	Stool exam	41.3	Nyantekyi et al., 2014
11		2015	374 schoolchildren	*Schistosoma mansoni*<br>Soil-transmitted helminths<br>*Ascaris lumbricoides*<br>*Trichuris trichiura*<br>Hookworms	Formol-ether concentration	31%<br>52.4%<br>44.4%<br>11%<br>7.7%	Tadege and Shimelis 2017
12		2015	302 School-aged children	Soil-transmitted helminths<br>*Ascaris lumbricoides*<br>*Trichuris trichiura*	Kato-Katz	41.7%.<br>19.8%<br>15.6%	Emana et a., 2015
13			393 schoolchildren	*Giardia duodenalis*<br>*Cryptosporidium* spp	RT-PCR	55%<br>4.6%	De Lucio et al., 2016
14		2001-2016	National	Malaria		4.31% to 2.9%	Taffese et al., 2018
15	KENYA	2012/2014	Pre-school aged children	*Schistosoma mansoni*	Kato-Katz	18%	Won et al., 2017

			3612 samples		Antibody response	50%	
16			285 HIV patients	*Entamoeba histolytica*/dispar<br><br>*Giardia lamblia*<br><br>*Entamoeba coli*	Fresh stool wet mounts; Modified ZN; Formol-ether conc.	58.3<br><br>16.6<br><br>5.9	Kipyegen et al., 2012
17		2001	1246 children 10−12 years	*Schistosoma mansoni*	Kato-Katz	16.3	Handzel et al., 2003
18		2012	4931 all age	Soil-transmitted helminths<br>Hookworms<br>*Ascaris lumbricoides*<br>*Trichuris trichiura*	Kato-Katz	32.7<br>16.7<br>17.9<br>6.1	Freeman et al., 2015
19		2012	21528 school	Soil-transmitted	Kato Katz	32.4	Mwandawiro et al.,

			children	helminths<br>Hookworms<br>*A. lumbricoides*<br>*T. trichiura*<br>*S. mansoni*<br>S. haematobium		 15.6<br>18.0<br>6.6<br>2.1<br>14.8	2013
20			692 pre-school & school children	Soil transmitted helminths<br>*Ascaris spp.*<br>*Trichuris spp.*<br>Hookworm	Kato Katz	40.5 & 40.7<br><br>24.1 & 22.7<br>24.0 & 28.8<br>< 0.1	Davis, et al. 2014
21	RWANDA	2010	109 students<br>Among infected:	Intestinal parasites<br>*E. histolytica*<br>*Trichomonas intestinalis* and *A. lumbruicoides*<br>*Giardia duodenalis*<br>*Ancylostoma duodenale*	Saline smear	50.5<br>54.5<br>20<br><br>3.6<br>1.8	Niyizurugero et al., 2013

22		2014/2015	Two districts	Intestinal worms and parasites in general		9997 cases	Stone and Ndagijimana 2018
23	SUDAN		300 <5-year children	Giardiasis<br>*Entamoeba histolytica*<br>*Entamoeba coli*	Stool exam	21.1<br>0.7<br>2.7	Karrar and Rahim 1995
24		2002	National estimate	Malaria	Hospital data + simulation	9 million episodes	Abdalla 2007
25	TANZANIA	2009	388 adult population	*Schistosoma mansoni*	Abdominal ultrasound	42.2	Kaatano et al., 2015
26		1996	Schoolchildren 284, 350	Schistosomiasis<br>• Urinary<br>• Intestinal		37, 86.3<br>22.9, 43.5	Poggensee et al., 2004
27		2010/2011	185 humans around national park	*Cryptosporidium spp.*		4.3	Parsons et al., 2015
28		2015/2016	2000 households	Malaria	Blood slide	13%	Khatib et al., 2018

			randomly selected		microscopy		
29	UGANDA		642 people	*Schistosoma mansoni*	Stool exam	81.6 (males)<br>81.3 (females)	Kabatereine et al., 1992
30			446 > 6 years old people	*Schistosoma mansoni*	Modified Kato-Katz	88.6	Tukahebwa et al., 2013
31		1996/1997	Fishing community	*Schistosoma mansoni*		72	Kabatereine et al., 2004
32			301 patients presenting with clinical signs	*Entamoeba histolytica*	Routine microscopy	19.93%	Ekou et al., 2012
33		2007	108 people living near national park	*Giardia duodenalis*	Stool RT-PCR	40.7%	Johnston et al., 2010
34	SOMALIA		General population in two localities	Schistosomiasis (urinary)		27.2% and 58.1%	Arfaa F 1975
35			Farmers from many parts	*Schistosoma haematobium*		65·8%, 48·9% and 60·;7%	Koura et al., 1981

36			Two institutions	Soil-transmitted helminths or protozoa		About 85%	Ilard et al., 1987
37	MAURITIUS		50 schools	*Schistosoma haematobium*		15% - >50%	Cowper 1953
38	ERITREA		Country average	*Schistosoma mansoni*		2.4%	Ministry of Education, 2002
39			Country average	*Schistosoma mansoni*		5%	Lai et al., 2015
40			75 vegetable farmers using wastewater irrigation	Giardiasis		45%	Srikanth amd Naik 2004
41	MADAGASCAR	2015	1958 school-age children	*Schistosoma haematobium*<br>*Schistosoma mansoni*<br>Soil-transmitted helminths	Kato-Katz	30.5 %<br>5.0 %<br>4.7 %.	Rasoamanamihaja etal., 2016

42			399 school-age children	*Schistosoma haematobium*<br><br>*Schistosoma mansoni*	Urine filtration<br><br>Circulating cathodic antigen (CCA) and Kato-Katz	0<br><br>93.7%	Spencer et al., 2017
43		1999-2017	Eritrean migrants	Malaria	Hospital records	146 cases	Schlagenhauf et al., 2018
44	SEYCHELES	1988		*T trichiura,*<br>Hookworms<br>*Ascaris lumbricoides,*<br>*Giardia intestinalis*<br>*Entamoeba histolytica*	84%<br>22%<br>8%<br>6%<br>5%		Kitua et al., 1988
45				*Ascaris lumbricoides*<br>*Trichuris trichiura*	17.7%<br>53.3%		Albonico et al., 1996

				Hookworms<br>*Strongyloides stercoralis*<br>*Entamoeba histolytica*<br>*Giardia intestinalis*	6.3%<br>1.1%<br>4.6%<br>3.3%		
46		2010-2016	Hospital uncomplicated malaria	Malaria	1.4 - 2.0%		Ihantamalala et al., 2018
47	DJIBOUTI	1998-2009	Hospital febrile cases	Malaria	<5%	Blood slide microscopy	Ollivier et al., 2011
